# Individuality in music performance: introduction to the research topic

**DOI:** 10.3389/fpsyg.2014.00661

**Published:** 2014-06-25

**Authors:** Bruno Gingras

**Affiliations:** Department of Cognitive Biology, Faculty of Life Sciences, University of ViennaVienna, Austria

**Keywords:** music performance, individuality, individual differences, piano performance, vocal emotions

The ability to discriminate among individuals is crucial in species, such as humans, that place a premium on kin recognition (Tang-Martinez, 2001). Identity cues used by humans comprise not only visual cues, including relatively static cues such as facial features (Carey, 1992) or dynamic displays such as gait and walking (Blake and Shiffrar, 2007), but also auditory cues such as voices (Belin et al., 2004), clapping patterns (Repp, 1987), or even tones which follow temporal patterns similar to clapping (Flach et al., 2004).

Cues to individuality can also be communicated efficiently through music. Indeed, along with emotion and structural cues, artistic individuality seems to be a key element conveyed in music performance. Over the last few decades, a growing body of research has examined issues related to individuality in musical performance (e.g., Repp, 1992; see Sloboda, 2000 for a review). Yet, the means by which individuality is musically expressed and perceived have remained poorly elucidated until recently. Hence, the aim of this Research Topic is to provide a forum for interdisciplinary research broadly centered on individuality and individual differences in music performance. This goal was successfully achieved, and the 14 contributed articles illustrate the depth and breadth of the topic, with themes ranging from personality correlates of flow proneness among pianists to unique “fingerprints” in the singing voice.

Setting the tone for the Research Topic, Wöllner (2013) emphasized in an opinion piece the importance of using averaged features, representing the mean of a large sample of performances by different performers, rather than computer-generated “deadpan” reproductions as the baseline for quantifying individuality in music performance. On a related issue, Farbood and Upham (2013) compared listener judgments of musical tension obtained for a recording of a Schubert song and its computer-generated harmonic reduction, showing that differences in perceived tension changes between the two excerpts highlighted interpretive choices in performance.

Historically, a substantial body of music performance research has focused on piano performance (see Gabrielsson, 2003 for a review), and this trend was maintained here. Van Vugt et al. (2013) explored the individuality associated with small but systematic temporal deviations in musical scales played by pianists, showing that although human listeners were not able to distinguish these “temporal fingerprints” by ear, high accuracy rates were obtained by classifiers. Bernays and Traube (2014) investigated individuality in pianists' performance of timbral nuances, and their analysis revealed that pianists exhibited unique profiles associated with different sonorities, while at the same time displaying common patterns of dynamics and articulation for each timbral color. Marin and Bhattacharya (2013) identified emotional intelligence and amount of daily practice as predictors of individual differences in proneness for flow among pianists, but did not observe a correlation between flow and high achievement in piano performance. Their study was the object of a commentary by Srinivasan and Gingras (2014) exploring the putative role of control and attention in flow states in music performance.

Two articles focused on the harpsichord, another keyboard instrument that, unlike the piano, has been relatively neglected so far in music performance research. Gingras et al. (2013) invited harpsichordists to record three different pieces and identified global markers of individuality, such as performers consistently using a more detached articulation across all three pieces, as well as associations between the note-by-note expressive profiles of different performers that subsisted across pieces or expressive parameters. In a follow-up to an earlier study on organ performance (Gingras et al., 2011), Koren and Gingras (2014) investigated whether listeners could reliably identify harpsichordists playing short excerpts from two different pieces. They found that musicians were more accurate than non-musicians, and only musicians performed above chance when matching the two different pieces to the same performer.

Voice production and perception was a major area of interest, with five contributions. Hutchins and Moreno (2013) proposed a new model to account for the variability between vocal perception and performance abilities in the general population. Their Linked Dual Representation model, which posits that vocal information can be encoded either as a symbolic or as a motoric representation, leads to a series of intriguing predictions about speech imitation, singing, and response timing. In a similar vein, Yang et al. (2013) investigated the coexistence of perceptual pitch deficits with pitch production deficits in music and in Mandarin speech in both amusics and tone agnosics, and their results suggest that the perception-production relationship for pitch among individuals may be domain-dependent. Trehub et al. (2013) confirmed the presence of individual cross-modal signatures in maternal speech and singing which can be discerned by both adults and infants, enabling listeners to successfully link recordings of unfamiliar speaking or singing voices to silent videos of the talkers or singers. Two articles focused more specifically on emotional singing: Quinto et al. (2014) examined the use of facial movements to communicate emotion, confirming the central role of facial expressions in vocal emotional communication while at the same time highlighting individual differences between singers, while Livingstone et al. (2014) analyzed the influence of vocal training and acting experience on the perception of vocal quality and emotional genuineness. They reported that acting experience was associated both with a decrease in voice quality and with an increase in perceived genuineness.

Finally, two studies addressed applied research topics related to individual differences in music performance. Williamon et al. (2014) designed and tested two simulated performance environments to help performers cope with issues related to performance anxiety, and discussed potential implications for performance training. Fritz et al. (2013) showed that participants' mood during exercise machine workout is enhanced more strongly with individualized musical feedback modulated by the participants' movements than with passive music listening.

In summary, this Research Topic both confirms and extends earlier findings, while at the same time opening up new avenues of research, especially in keyboard and voice performance. More generally, it highlights the cross-fertilizing potential of applying a multidisciplinary approach to the study of individuality in music performance, emphasizing the importance of fostering collaborations among musicologists, computer scientists, psychologists, neuroscientists, and the performers themselves.

## Conflict of interest statement

The author declares that the research was conducted in the absence of any commercial or financial relationships that could be construed as a potential conflict of interest.
